# Dynamic interaction of genetic risk factors and cocaine abuse in the background of Parkinsonism – a case report

**DOI:** 10.1186/s12883-019-1496-y

**Published:** 2019-10-28

**Authors:** Anett Illés, Péter Balicza, Viktor Molnár, Renáta Bencsik, István Szilvási, Maria Judit Molnar

**Affiliations:** 1 Institute of Genomic Medicine and Rare Disorders, Semmelweis University, Budapest, Hungary; 2 Department of Nuclear Medicine, Hungarian Defence Force Medical Center, Budapest, Hungary

**Keywords:** Parkinson’s disease, Parkinsonism, cocaine, *LRRK2*, Genetic risk factor

## Abstract

**Background:**

Parkinsonism is a complex multifactorial neurodegenerative disorder, in which genetic and environmental risk factors may both play a role. Among environmental risk factors cocaine was earlier ambiguously linked to Parkinsonism. Former single case reports described Parkinsonism in chronic cocaine users, but an epidemiological study did not confirm an increased risk of Parkinson’s disease. Here we report a patient, who developed Parkinsonism in young age after chronic cocaine use, in whom a homozygous *LRRK2* risk variant was also detected.

**Case presentation:**

The patient was investigated because of hand tremor, which started after a 1.5-year period of cocaine abuse. Neurological examination suggested Parkinsonism, and asymmetrical pathology was confirmed by the dopamine transporter imaging study. The genetic investigations revealed a homozygous risk allele in the *LRRK2* gene. After a period of cocaine abstinence, the patient’s symptoms spontaneously regressed, and the dopamine transporter imaging also returned to near-normal.

**Conclusions:**

This case report suggests that cocaine abuse indeed might be linked to secondary Parkinsonism and serves as an example of a potential gene-environmental interaction between the detected LRRK2 risk variant and cocaine abuse. The reversible nature of the DaTscan pathology is a unique feature of this case, and needs further evaluation, whether this is incidental or can be a feature of cocaine related Parkinsonism.

## Background

Pathomechanism of Parkinson’s disease (PD), which is the second most common neurodegenerative disorder, is defined by the progressive loss of dopaminergic neurons in the substantia nigra [1]. The dopaminergic system plays an important role in several vital mechanisms, such as movement control, cognition and controlling reward. For the normal functioning of dopaminergic neurons the dopamine reuptake is essential from the synaptic cleft into presynaptic neurons via dopamine transporters [2]. There are several drugs (such as cocaine) causing increased extracellular level of dopamine resulting in euphoric effects and motoric symptoms [3].

In case of PD, there are evidences, which suggest that dopamine transporter (DAT) dysregulation is also a factor in the disease mechanism [4]. Cocaine enhances dopaminergic signalling as it binds to DAT and blocks the reuptake of dopamine from the synaptic cleft [5]. The suspected association between cocaine abuse and the increasing risk of PD was previously described in several patients [6] although there are several cases where despite cocaine abuse no correlation with PD was observed [7].

Although the evidences for higher risk of PD among cocaine users is controversial, it is already proved that the brain structure is altered and the conformation of alpha-synuclein become more compact [8]. In PD pathogenesis the misfolded alpha-synuclein plays crucial role in the death of dopaminergic neurons therefore in the progression of PD [9]. In cocaine abusers overexpression of alpha-synuclein has been described in dopaminergic neurons, potentially increasing the risk for degenerative changes in dopaminergic neurons [10]. As *SNCA* is one of the most common cause of PD there are possibility that other genetic risk factors, such as *PARK2*, *LRRK2*, *PINK1* and *DJ-1* [11], also could contribute to the development of PD even in early age due to the cumulated effect of cocaine abuse and genetic risk.

DAT-SPECT (dopamine transporter single photon emission computed tomography) imaging enables differentiation of neurodegenerative causes of Parkinsonism, from other movement or tremor disorders where typically the DAT-SPECT study will be normal. Impaired function of DAT is reducing striatal binding of DaTSCAN, however it is not specific to PD. Several cocaine analogues labelled with ^123^I sufficient for SPECT binds with high affinity to DAT [12]. The most common analogue in the clinical practice is ^123^I-FP-CIT (DaTSCAN, GE Healthcare) [12]. The method can measure either the DAT density on the presynaptic terminal, or nigrostrital fiber density.

In our case study we are discussing the association of the genetic and environmental factors in a cocaine user young patient with reversible Parkinsonian symptoms.

## Case presentation

A 44 years old male patient was referred to our neurogenetic outpatient clinic, for examination of hand tremor. In the past medical history gastroesophageal reflux disease (Los Angeles grade A), LIV-V discus herniation and Type I (Wenckebach) second-degree atrioventricular block was present. The latter caused no symptoms, and no medical intervention was necessary. The patient took pantoprazol regularly. Before the onset of the hand tremor the patient used nasal cocaine regularly for a period of 18 month. He used ~ 1 g/day (15 mg/kg) with nasal insufflation. During the cocaine use irritability and insomnia, dissociative symptoms such as depersonalization and derealisation, developed. Because of the latter he stopped cocaine use 10 months before the examination. He realized his hand tremor approximately 3–4 months after cessation of the drug.

**Fig. 1:**
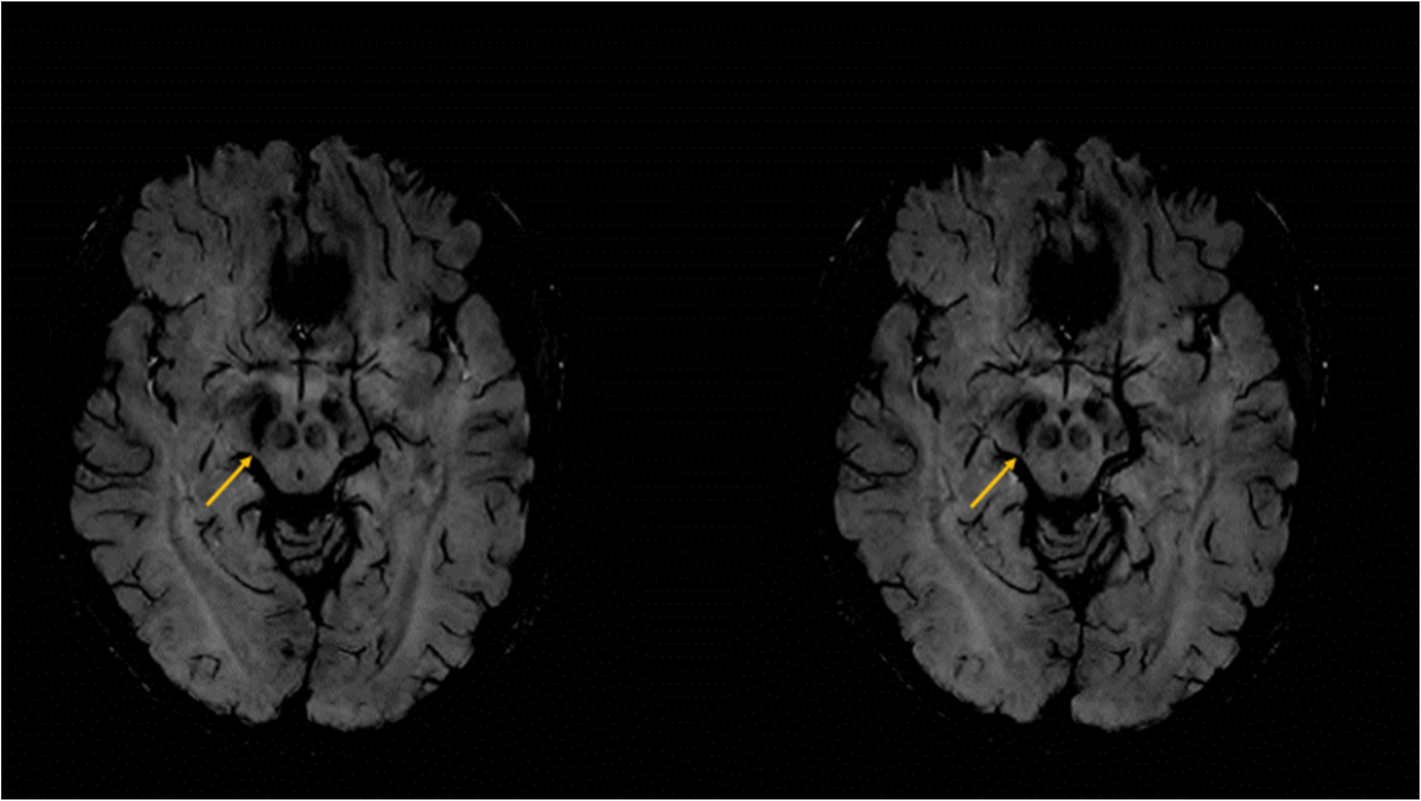
Brain MRI of the patient. On the axial susceptibility weighted images in the plane of the mesencephalon, the substantia nigra is identifiable both sides. The swallow tail sign is normally present in 3 T imaging at the area indicated by the arrows, but it is absent in the patient

**Fig. 2:**
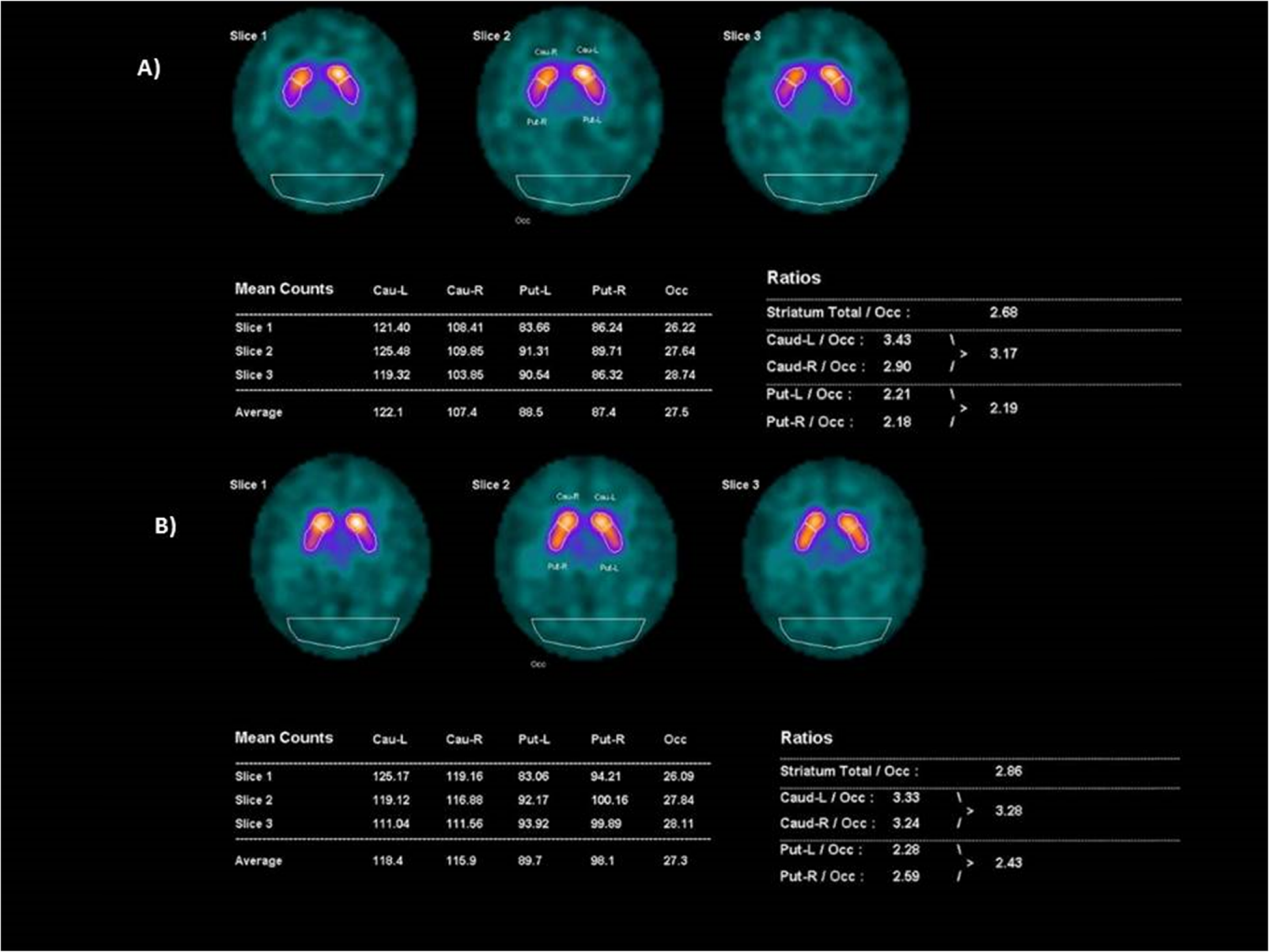
DaTscan of the patient in two time points. The Figure shows the radiopharmacon binding in the area of the basal ganglions. The measurements and calculated ratios for quantitative analysis in the volumes of interest are also listed. Figure **a** was taken after the first examination of the patient. At this time decreased radiopharmacon binding was present in the right striatum (mainly the caudate). Figure **b** was taken after 1 year of cocaine abstinence. At this time point, normal binding is detectable in the right caudate

During the neurological examination hand tremor affected asymmetrically the right hand more than the left hand, and was mainly postural increasing with holding small weights. In addition, signs of mild Parkinsonism (mild bradykinesia and rigor in the right hand) was also detected. Altogether, the neurological examination suggested incipient Parkinsonism, but the tremor was atypical (not resting type). According to the MDS classification of tremors [13] it was classified as isometric tremor syndrome. From the family history, it is noticeable that the father of the patient suffered from postural hand tremor in older age. The son of our patient was examined because of restless leg syndrome at age 13 years. Routine blood studies were normal, including copper, ferritin and ceruloplasmin. Abdominal ultrasound was normal. Brain MRI (3 T) showed no structural or vascular lesions, basal ganglia were normal, but absence of swallow tail sign was detected (Fig. 1), suggesting Parkinson’s disease. For further clarification DaTscan was performed by a double-headed SPECT system (GE Infinia II with Xeleris workstation) using a standard acquisition protocol according to the EANM guideline [12] with 170 MBq I-123-Ioflupane tracer. This tracer has a high affinity not only to DAT but to the serotonin transporter (SERT) and norepinephrine transporter (NET) as well [14]. However, the concentration of DAT in the basal ganglia is much higher, than that of the other transporters, therefore its measure is appropriate for DAT. The striatal binding was evaluated both by semiquantitative visual evaluation and for a more accurate comparison the DaTQUANT software (created by GE Healthcare in 2013 adapted in 2015, as a quantitative binding method with normal database) has been used [15]. It showed asymmetrically decreased radiopharmacon binding on the right side in the caudate nucleus 3.0 h after intravenous injection of the I-123-Ioflupane (Fig. 2**/**a). During the time of the DaTscan no cocaine use has been reported. Although we had only self-report about the cocaine use, the long-term observation of the patient and the close follow-up with a good compliance, and the improvement of the clinical symptoms convinced us about the reliability of the anamnestic data.

For the genetic investigation DNA was isolated from blood with QIAamp DNA blood kit, according to the manufacturer’s protocol (QIAgen, Hilden, Germany). Sanger sequencing was performed in the whole coding region and exon/intron boundaries of *SNCA*, *PARK2* and *PINK1*, *LRRK2* gene by using ABI Prism 3500 DNA Sequencer (Applied Biosystems, Foster City, USA). Exonic copy number variations were analyzed by multiplex ligation-dependent probe amplification (MLPA, SALSA MLPA Kit P051-D1 Parkinson; MRC Holland, Amsterdam, The Netherlands). In the *LRRK2* gene a homozygous risk factor variant, NM_198578.4:c.4939 T > A, p.Ser1647Thr (codon change: TCA > ACA) was detected [16]. The segregation analysis detected the S1647 T *LRRK2* variant in heterozygous state in the parents of the patient. MLPA did not detect any exonic copy number variations. Based on the clinical and DaTscan findings we suspected Parkinson syndrome, associated with a toxic-genetic interaction. Selegiline was prescribed, but the patient omitted the medication, because the tremor was worsening from the drug. After 1 year of cocaine abstinence the tremor significantly decreased. One and half year after the first DaTscan a control investigation was performed, and showed normal radiopharmacon binding in the striatum, with only mild asymmetry. The right caudate binding returned into the normal range, and the right striatal binding was higher than at the first examination (Fig. 2**/**b).

## Discussion and conclusions

Cocaine use is associated with a range of movement disorders [3], and has complex effects on the central nervous system. Possible ways to categorize these effects is based on time characteristics, i.e. neurologic complications with acute or chronic use, or whether the patient is an active user, early or late abstinent. The main acute pharmacological effect of cocaine is dopamine (DA) reuptake inhibition, which elevates synaptic DA levels.

Literature information about cocaine’s effect on dopamine transporter (DAT) level expression in human is scarce and available information from experimental animal studies are also contradictory at times. There are two possible mechanism supported by the literature, by which we tried to interpret our findings, i.e. the low DAT binding, which later normalized.

On one hand, in response to the elevated DA levels, DAT downregulation might take place, as a compensatory mechanism [17]. This compensatory mechanism decreases the acute DA elevation with the use of cocaine, but on the long term it leads to DA deficiency in the caudate nucleus and frontal cortex as DA synthesis and reuptake is both needed for synaptic storage [18]. In acute cocaine abstinence the DATs start to upregulate as shown by other DaTscan studies [19]. This might explain our results, why we have seen decreased DAT binding, which later normalized. In this scenario we hypothesize that DaTscan in our patient was performed in a time window when DAT levels are still decreased; however, the patient was already abstinent. As an acute withdrawal symptom decreasing DA level results in psychological symptoms, restlessness and tremor [20]. Long term use of cocaine however also results in DAT decrease, and this might explain Parkinsonian features in abstinence as a result of DA depletion.

On the other hand, other studies in the literature suggest, that cocaine increases DAT expression, and abstinence of cocaine intake for a prolonged period of time decreases DAT level [5]. In this scenario, we can hypothesize that we have seen the decreased DAT-binding, because the patient was already abstinent for a long time, and this change in the expression later normalized.

It should be mentioned that the above described mechanisms are speculative and the effects of cocaine on the nervous system is complex. We also need to consider changes in D2 receptor expression [21] and possible long-term structural damage to dopaminergic synaptic terminals [18]. Effects might be dose and formulation dependent, as neurologic complications are more common with the smokable alkaloidal form of cocaine, known as „crack” [22]. Acute blood pressure elevations and cerebral vasospasm might also cause cerebrovascular events, such as acute ischemic stroke, or aneurysm rupture [23], but small subclinical ischemic events may also cause structural damage in the brain. Chronic cocaine abuse lead to increased age-dependent temporal lobe cortical atrophy [24], and decreased frontal white matter connectivity [25] shown by imaging studies.

The association of cocaine use with Parkinsonism is nevertheless complicated, and the literature information is scarce. On one hand, the acute elevation of synaptic DA levels may ameliorate “off” periods in Parkinson’s disease patients [26]. On the other hand chronic use was associated with Parkinsonian features in many case reports [20], although this was not confirmed by the epidemiological study of Callaghan et al. [27]. The above described mechanism suggests a pharmacological, reversible form of secondary Parkinsonism in our case. However, a further possible, non-pharmacologic link between Parkinsonism and chronic cocaine use might exist. Chronic cocaine exposure triggers alpha-synuclein overexpression [10], which might be an acute protective mechanism against increased oxidative stress, but which eventually lead to formation of Lewy bodies (LBs), and accelerated neurodegeneration. Besides, cocaine also physically binds to alpha-synuclein, which might cause deleterious conformational changes [8]. However, it is not probable, that these changes will cause reversible pathology on the DaTscan.

The long-term cocaine use has not the same effect as dopamine receptor blocking agents - DRBA, however these can induce also parkinsonism. Drug-induced Parkinsonism (DIP) should resolve after the causative agent has been withdrawn. Lim et al. [28] reported that Parkinsonism might persist for more than 6 months after discontinuation of the DRBA, and DaTscan showed normal striatal dopamine transporter binding at that time. Nine months after the discontinuation of the dopamine receptor blocking agent, Parkinsonism was significantly improved in their patients but not completely resolved [28].

In a number of patients, with DIP symptoms persist or may even worsen over time, suggesting the development of concomitant PD. There are speculations that the possible neurotoxic effect of neuroleptics exerted on a susceptible dopaminergic system would lead to a progressive process. To which extent a personal susceptibility plays a role remains to be determined and further genomic studies in patients exposed to neuroleptics who develop DIP or PD could eventually identify a genetic background of susceptibility [29]. Even if the pathomechanism is not the same in the cocaine induced Parkinsonism and DIP the personal susceptibility can be an important factor.

In our case the PD associated genes were investigated since the patient has movement disorders in his family. We detected only one genetic risk variant, which was previously associated with PD. The presence of this homozygous *LRRK2* polymorphism (S1647 T) has a very mild association with PD, with a low odds ratio (in our cohort OR: 1.787, 95%, CI: 0.8052 to 3.96 – Illes et al., unpublished data). In the presence of this genetic risk variant, even in homozygous status, appearance of Parkinsonism is not likely, but hypothetically in the presence of some environmental factors, which may influence dopamine level it may present itself. Similar mechanism was suggested by Lin et al. [30] in a Taiwanese population, where the S1647 T variant was only associated to Parkinsonism when environmental exposures were included in the logistic regression model. Further published studies indicated also significant interactive effects between environmental factors and genetic variants [31]. These kind of interactions are well described in the case of the serotonin transporter polymorphism association with depression [32]. However, it should be kept in mind that proof of the additional effect of *LRRK2* S1647 T polymorphism and cocaine abuse goes beyond the framework of our case study.

It is interesting, that in our patient, the MRI already showed some structural changes (absence of the swallow tail sign), indicating the damage of nigrostriatal pathway, and thus the acute pharmacological effect of cocaine might be also altered. The family history of hand tremor in the father and restless-leg syndrome in the child also suggest some already existing non-pharmacologic risk at the patient.

In summary, this case report may raise the possibility of a gene-environment interactions in the background of our patient’s symptoms. Our result suggests that some of these effects in the early state might partially reversible, as after a period of abstinence the patient’s Parkinsonian symptoms resolved. However, the patient needs longitudinal follow-up, as PD might later reoccur, as the consequence of the chronic effects of cocaine, and the additive effects of the *LRRK2* alteration. Further studies of S1647 T alteration and environmental interaction in a larger Hungarian cohort and functional studies in in vivo models are warranted to validate our hypothesis.

## Data Availability

The datasets used and/or analysed during the current study are available from the corresponding author on reasonable request.

## Abbreviations

CI: Confidence interval
DA: Dopamine
DAT: Dopamine transporter
DaTscan: Dopamine transporter single photon emission computerized tomography
DAT-SPECT: Dopamine transporter single photon emission computed tomography
DIP: Drug-induced Parkinsonism
DJ-1: Daisuke-Junko-1
DNA: Deoxyribonucleic acid
DRBA: Dopamine receptor blocking agents
LB: Lewy Body
LRRK2: Leucine-rich repeat kinase 2
MDS: Movement Disorder Society
MLPA: Multiplex ligation-dependent probe amplification
MRI: Magnetic resonance imaging
NET: Norepinephrine transporter
OR: Odds ratio
PARK2: Parkinson protein 2 E3 ubiquitin protein ligase
PD: Parkinson’s disease
PINK1: PTEN-induced putative kinase 1
SERT: Serotonin
SNCA: Alpha-synuclein
SPECT: Single-photon emission computed tomography
